# Copolymerization of Phenylselenide-Substituted Maleimide with Styrene and Its Oxidative Elimination Behavior

**DOI:** 10.3390/polym10030321

**Published:** 2018-03-15

**Authors:** Qian Liu, Xinghua Lv, Na Li, Xiangqiang Pan, Jian Zhu, Xiulin Zhu

**Affiliations:** State and Local Joint Engineering Laboratory for Novel Functional Polymeric Materials, Department of Polymer Science and Engineering, College of Chemistry, Chemical Engineering and Materials Science, Soochow University, Suzhou 215123, China; qliu1@stu.suda.edu.cn (Q.L.); t_zhuj@suda.edu.cn (X.L.); chemlina@suda.edu.cn (N.L.); xlzhu@suda.edu.cn (X.Z.)

**Keywords:** selenium-containing monomer, reversible addition-fragmentation chain transfer (RAFT), oxidation, carbon-carbon double bond

## Abstract

Selenium-containing monomer monophenyl maleimide selenide (MSM) was synthesized and copolymerized with styrene (St) using reversible addition-fragmentation chain transfer (RAFT) polymerization. Copolymers with controlled molecular weight and narrow molecular weight distribution were obtained. The structure of the copolymer was characterized by nuclear magnetic resonance, matrix-assisted laser desorption/ionization time-of-flight (MALDI-TOF) mass spectrum, Fourier transform infrared spectroscopy (FT-IR) and Ultraviolet–visible spectroscopy (UV-vis) spectroscopy. The copolymer can be oxidized by H_2_O_2_ to form carbon-carbon double bonds within the main chain due to the unique sensitivity of selenide groups in the presence of oxidants. Such structure changing resulted in an interesting concentration-related photoluminescence emission enhancement.

## 1. Introduction

The selenium containing group has been widely used as an antioxidant due to low binding energy of the selenium-carbon bond [1]. Many selenium-containing compounds are promising multi-responsive materials with unique redox properties [2]. Great efforts in material chemistry fields have been reported based on the redox property of selelnium-containing compounds [3,4,5]. Xu et al. reported a novel amphiphilic block copolymer with oxidation responsive selenide moieties which can undergo a disassembled in a mild oxidative environment and a reversible reduction upon the addition of Vitamin *C* [6]. In addition, depending on the redox property of selenium-containing compounds, some selenium-containing probes for sensitive and selective detection of oxygen species were reported [7]. For example, Manjare et al. designed a diselenide-based boron dipyrromethene (BODIPY) probe for detecting superoxide [8]. After oxidation, the ability for the photo-induced electron transfer from the selenium to the fluorophore was removed and the probe gave a fluorescence response. In 2017, a dynamic hydrogel containing diselenide moiety was proposed by Pan et al. which showed redox and temperature responses by selectively oxidation of selenol-terminated chain. Through this method, such material could lead to the reconstruction of a hyperbranched architecture and a transition to gel state [9].

In fact, selenium-containing moieties could be eliminated at mild conditions. Yuki et al. demonstrated that selenide end functionalized polymer could be oxidized to the corresponding polymer with terminal selenoxide, and they followed this method by elimination to give a polymer containing a double bond at terminal groups [10]. In 2016, Dai et al. obtained highly regioselective carbonselenation styrene in the presence of H_2_O_2_ [11]. This reaction is an efficient method for the synthesis of target alkenes via selenoxide *syn* elimination and chiral hydrocarbons that bear an aryl moiety at the stereogenic carbon atom if a chiral selenium regent is used.

Dithiomaleimide is a new fluorescent small molecule demonstrated by Rachel K. O’Reilly et al. in 2013 [12]. Additionally, in 2015, the same group presented a library of amino-maleimides which showed an intense fluorescence emission in cases of maleimide with ankyl primary amines substitution [13]. Recently, using a stable Ru(II)-catalyst, Mahiuddin Baidya reported a series of seleno-substituted maleimides with excellent yield (up to 94%) [14]. In 2016, Haddleton et al. synthesized a dithiomaleimide-containing monomer [15]. It was successfully copolymerized with poly(triethyene glycol methyl ether acrylate) to endow copolymers with thermos-responsive and fluorescent properties. Furthermore, polymers with multiple carbonyl (C=O) moieties can demonstrate strong photoluminescence (PL) emission under proper conditions [16,17]. These polymers showed poor PL emission in solution. However, in solids or viscous liquids, PL emission could be enhanced [18]. In 2017, Shiao-Wei Kuo et al. reported an interesting monomer, namely, 2-aminobutyl maleimide isobutyl polyhedral oligomeric silsesquioxane, which had a lack of conventional fluorescent groups [19]. After it copolymerized with maleimide-containing monomer, the copolymer displayed strong light emission due to the intermolecular hydrogen bonding between the amino and dihydrofuran-2,5-dione group and clustering of the locked C=O groups.

Seleno-mediated radical polymerization (SeRP) has been widely used as a method applicable to get selenium-containing polymers [20,21]. However, there have been few reports about the radical polymerization of vinyl monomers containing selenide moiety. This was probably due to the strong interaction of selenium stom with free radicals [22,23]. In 1996, Yuki et al. reported a new type of selenium-containing vinyl monomers (*p*-methylselenostyrene and *p*-phenylselenostyrene) which can polymerize with azodiisobutyronitrile (AIBN) or benzoyl peroxide (BPO) as initiator to afford the corresponding polymers [24]. In 2017, Pan et al. demonstrated a selenide-containing monomer to the preparation of dynamic covalent cross-linked polymers [25]. This monomer can simultaneously act as a comonomer, cross-linker, initiator and mediator.

**Scheme 1:**
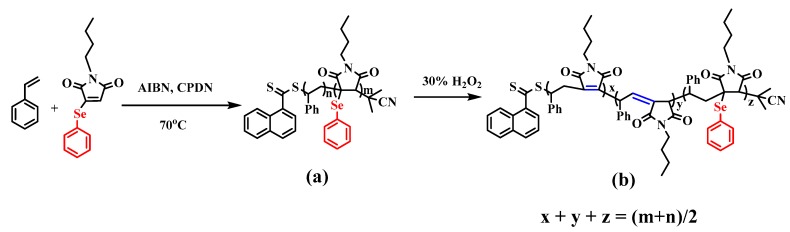
Synthetic route of copolymer (**a**) and its corresponding oxidative structure (**b**).

Based on the literature survey, herein, we prepared a selenium-containing maleimide monomer monophenyl maleimide selenide (MSM) which could copolymerize with styrene by reversible addition-fragmentation chain transfer (RAFT) polymerization (Scheme 1a). Copolymers with controlled molecular weight distribution were successfully obtained. Furthermore, due to the cleaving of weak selenium-carbon bond, the molecular of copolymer was decreased when treated with H_2_O_2_ and then carbon-carbon double bonds were formed in the backbone of oxidized copolymer (Scheme 1b). This procedure provides a new candidate for preparing cross-linked polymer using the internal carbon-carbon double bonds as cross-linker. Besides, for the oxidized copolymer, the PL emission intensity of spectral region at 460nm could be enhanced both in solid state and in solution.

## 2. Materials and Characterization

### 2.1. Materials

Styrene (St, Sinopharm Chemical Reagent, Shanghai, China, 99%) was purified by passing through a short Al_2_O_3_ columns before use. Diphenyl diselenide (DPDS) [26], 2-cyanoprop-2-yl-1-dithionaphthalate (CPDN) [27,28] and *N*-butyl-monobromomaleimide [29] were synthesized according to literature procedures. 2,2-Azobisisobutyronitrile (AIBN, Sinopharm Chemical Reagent, China, 98%) was purified by recrystallization from ethanol. Other reagents were purchased from Sinopharm Chemical Reagent Co., Ltd. and were used without further purification.

### 2.2. Characterization

The number-average molecular weight (*M*_n_) and molecular weight distribution (*Đ*) of the polymers were determined by a TOSOH HLC-8320 gel permeation chromatograph (GPC) equipped with a refractive index and UV detector (Tosoh Bioscience Shanghai Co. Ltd., Shanghai, China), using two TSK gel super 20 Multipore HZ-N (4.6 × 150 mm, 3 μm beads size) with measurable molecular weights ranging from 500 to 1.9 × 10^5^ g/moL. Tetrahydrofuran (THF) was used as the eluent at a flow rate of 0.35 mL/min at 40 °C. Data acquisition was performed using EcoSEC software (Tosoh Bioscience Shanghai Co. Ltd., Shanghai, China). The ^1^H NMR and ^13^C NMR spectra were recorded on a Bruker 300 MHz nuclear magnetic resonance instrument (Bruker Daltonics Inc., Billerica, MA, USA) using CDCl_3_ as the solvent and tetramethylsilane (TMS) as the internal standard. The ^77^Se NMR was recorded on a Bruker 600 MHz nuclear magnetic resonance (Bruker Daltonics Inc., Billerica, MA, USA) instrument using CDCl_3_ as the solvent and tetramethylsilane (TMS) as the internal standard. Matrix-assisted laser desorption/ionization time-of-flight (MALDI-TOF) mass spectroscopy were acquired on an UltrafleXtreme MALDI TOF mass spectrometer (Bruker Daltonics Inc., Billerica, MA, USA) equipped with a 150 kHz smart beam-II laser. FT-IR spectra were obtained on a BrukerTENSOR-27 FT-IR spectrometer (Bruker Daltonics Inc., Billerica, MA, USA) by mixing the polymer with KBr as tablets. The UV-vis spectra were recorded on a Shimadzu UV-3150 spectrophotometer at room temperature (Shimadzu China, Shanghai, China). The photoluminescence (PL) spectra of the samples were determined with a Hitachi F-4600 fluorescence spectrophotometer (Hitachi (China) Ltd., Shanghai, China) using a monochromated Xe lamp as an excitation source with a scanning rate of 1200 nm min^−1^. The excitation and emission slits were both set at 5 nm. Differential scanning calorimetry (DSC) was performed with heating and cooling rates of 10 °C/min on a PerkinElmer Diamond DSC instrument (PerkinElmer, Billerica, MA, USA) equipped with a cooling system under continuous nitrogen flow. Thermogravimetric analysis (TGA) was performed on a 2960 SDT TA instruments with a heating rate of 10 °C/min from room temperature to 800 °C under a nitrogen atmosphere.

### 2.3. Synthesis of Monoselenomaleimide (MSM)

To a solution of DPDS (1.56 g, 5 mmol) in 50mL C_2_H_5_OH-DMF (3:2) was added NaBH_4_ (0.15 g, 4 mmol) under nitrogen atmosphere at room temperature. After 30 min of stirring, *N*-butyl-monobromomaleimide (1.16 g, 5 mmol) was added at 0 °C. The solution was stirred for 30 min. The reaction mixture was extracted with dichloromethane (3 × 40 mL) and washed with water. The organic layer was dried over anhydrous magnesium sulfate and concentrated. The residue was subjected to column chromatographic separation (Silica gel, [Hexane]/[Ethyl acetate] = 20/1, *v/v*) to give 0.81g (66% yield) of monophenyl maleimide selenide (MSM). ^1^H NMR (300 MHz, CDCl_3_, δ, ppm) (Figure S1): 7.66–7.64 (m, 1H), 7.63–7.61 (m, 1H), 7.50–7.38 (m, 3H), 5.86 (s, 1H), 3.51 (t, *J* = 7.2 Hz, 2H), 1.63–1.50 (m, 2H), 1.31 (m, 2H), 0.92 (t, *J* = 7.3 Hz, 3H). ^13^C NMR (75 MHz, CDCl_3_, δ, ppm) (Figure S2): δ = 169.76 (1C), 169.01 (1C), 150.22 (1C), 135.88 (2C), 130.26 (2C), 130.04 (1C), 125.48 (1C), 123.90 (1C), 38.01 (1C), 30.63 (1C), 19.96 (1C), 13.55 (1C). ^77^Se NMR (600 MHz, CDCl_3_, δ, ppm) (Figure S3): δ = 387.51 (1Se).

### 2.4. General Procedures for Reversible Addition-Fragmentation Chain Transfer (RAFT) Polymerization of Styrene and MSM

The polymerization was carried out in a baked Schlenk tube under argon protection. As for the molar ratio with [St]_0_/[MSM]_0_/[CPDN]_0_/[AIBN]_0_ = 200/200/2/1, MSM (2.67 g, 8.62 mmol), St (1.0 mL, 8.63 mmol), CPDN (23.3 mg, 0.086 mmol) and AIBN (7.1 mg, 0.043 mmol) were added into the tube. Toluene (50 µL) was added and used as internal standard for ^1^H NMR calculation. A small portion of the mixture was collected for the determination of the initial monomer ratio by ^1^H NMR. The solution was degassed through a freeze-pump-thaw three times. Then, the reaction tube was bathed in an oil bath preset at 70 °C. Monomer conversion was determined by ^1^H NMR spectroscopy. After the predetermined time, a portion of the polymerization mixture was directly dissolved in CDCl_3_ for ^1^H NMR analysis to determine the respective monomer conversion. In the whole process of polymerization, the contents of toluene did not change, so the integral of toluene in the ^1^H NMR could be easily used as an internal standard for evaluating the conversion of monomers by comparing the integral value of double bonds with that of toluene. The typical spectrum is shown in Figure S4. The residual mixture was dissolved in THF and precipitated in an excess of methanol. After filtration, the copolymer was dried in vacuum at 30 °C for 24 h.

### 2.5. Oxidation of Copolymer Using H_2_O_2_ as Oxidizing Agent

The oxidation was carried out in a 2 mL ampoule with copolymer (35 mg, sample in Table 1 Entry 4) in THF (1 mL) and oxidized with H_2_O_2_ (45 µL, 30% *v/v*) at room temperature for 3 h. Subsequently, the solution was precipitated in methanol. After filtration, the copolymer was dried in vacuum at 30 °C for 24 h.

## 3. Results and Discussion

### 3.1. Copolymerization of St and MSM

Maleimide is a well-known electron deficient monomer, which has showed poor homopolymerization ability and has favored copolymerization with an electron-rich monomer. Herein, the selenide substituted maleimide, MSM, was copolymerized with styrene. RAFT polymerization was used for the aim of synthesis polymers with controlled molecular weight along with narrow molecular weight distribution. The polymerization results under different molar ratios of St to MSM were summarized in Table 1. As shown in Table 1, copolymerization of St and MSM could be carried out under RAFT polymerization using CPDN as the RAFT agent and AIBN as the initiator at 70 °C. Polymers with narrow molecular weight distribution could be obtained. However, the molecular weight distribution (*Đ*) of the final copolymer increased with the decreasing of [St]_0_/[MSM]_0_ molar ratio, which may due to the reactivity of selenide with radical [30]. Furthermore, in case of no comonomer St (Table 1 Entry 5), no polymer was obtained even after 24 h of polymerization time, indicating that MSM is difficult to homopolymerize.

**Figure 1:**
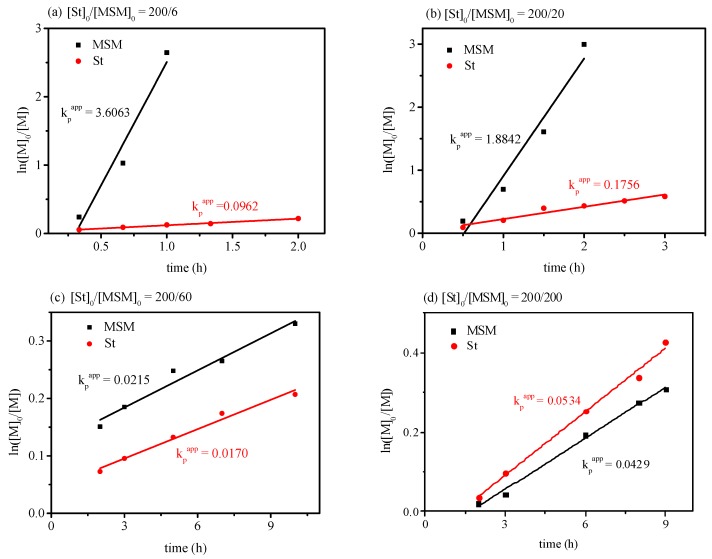
Kinetic plots of reversible addition-fragmentation chain transfer (RAFT) copolymerization of St and MSM with different molar ratios, (**a**) [St]_0_/[MSM]_0_ = 200/6; (**b**) [St]_0_/[MSM]_0_ = 200/20; (**c**) [St]_0_/[MSM]_0_ = 200/60; (**d**) [St]_0_/[MSM]_0_ = 200/200, *V*_St_ = 1.0 mL, *V*_toluene_ = 50 µL, temperature 70 °C.

To deeply understand the RAFT copolymerization behavior of St and MSM, the polymerization kinetics with different molar ratios were investigated. The results were showed in Figure 1. The results showed that all the polymerization kinetics conveyed linear plots. Such results indicated that the propagation radical concentration remained approximately constant during the polymerization period. The “living”/controlled feature can also be manifested by other results. Figure S5 (number-average molecular weight and molecular weight distribution (*Đ*) vary with the overall monomer conversion) showed good linear relationships between molecular weight and overall monomer conversion. The GPC curves of the obtained polymers at different feeding ratios (Figure S6) exhibited unimodal profiles in all cases, confirming that the resulting polymers are generated by the copolymerization of MSM and St, rather than being a mixture of two homopolymers. It could be observed that, in general, the consumption rate of MSM increased with the increase of [St]_0_/[MSM]_0_ molar ratio.

**Figure 2:**
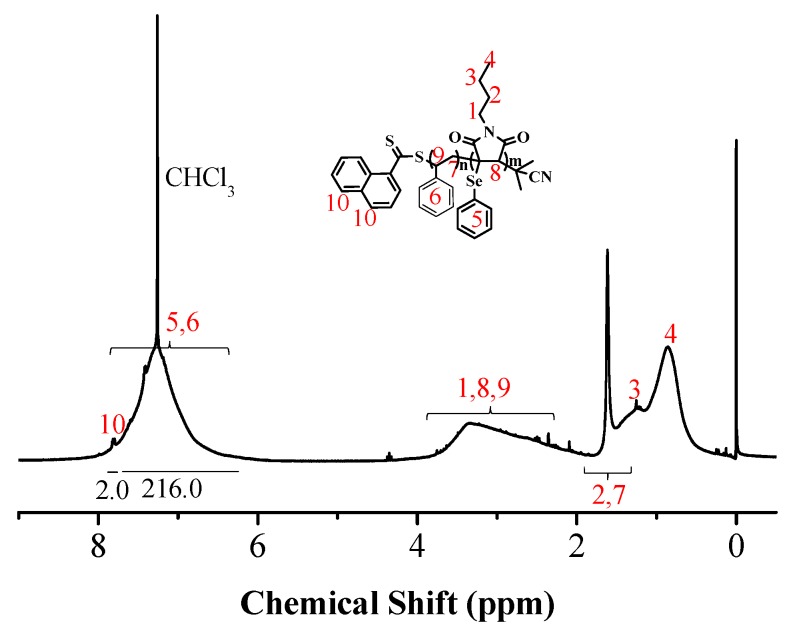
^1^H NMR spectrum of the RAFT copolymer in CDCl_3_.

**Figure 3:**
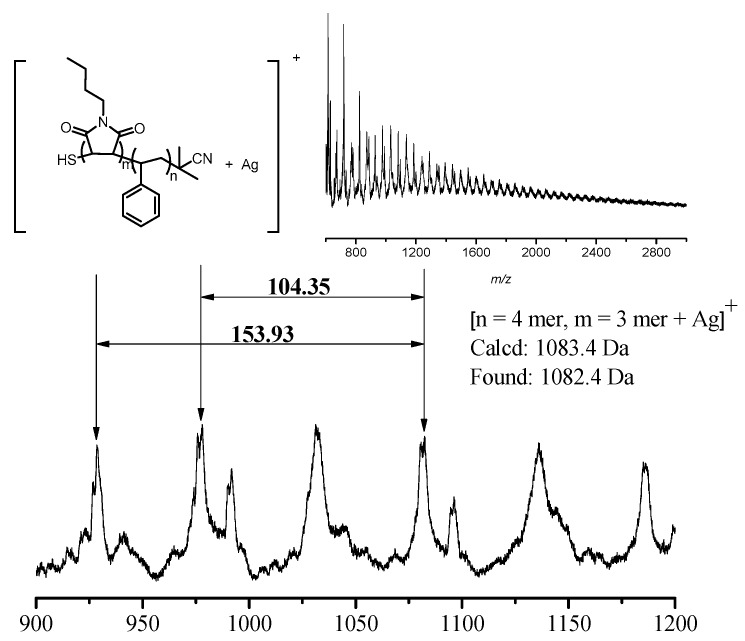
Expanded MALDI-TOF mass spectrum of the RAFT copolymer by using Ag salt as the cationization agent and a DCTB matrix.

The structure of the polymer ([St]_0_/[MSM]_0_/[CPDN]_0_/[AIBN]_0_ = 200/200/2/1, precipitated in methanol, *M*_n_ = 4600 g/mol, *Ð* = 1.47) was characterized by ^1^H NMR, ^13^C NMR, MALDI-TOF mass and FT-IR. From the Figure 2, the signals at about *δ* = 2.50–3.40 ppm could be ascribed to the protons adjacent to the nitrogen atom, protons from the fiveämembered ring, and protons from styrene monomeric unites adjacent to maleimide monomeric unites. The copolymer showed the signals at about 7.77–7.85 ppm (2H, integration = *I*_7.77–7.85_ = 2.0), which were ascribed to the protons at the chain end derived from CPDN. The signals at about 6.50–7.75 ppm (10H, integration = *I*_6.50–7.75_ = 216.0) were ascribed to the protons of benzyl from styrene monomeric unites and protons of phenyl-selenide groups. Based on the Figure 1d, the monomer ratio was 1:1 and the monomer conversion rates were close to each other. It can be assumed that the repeat units of St and MSM monomer are the same in the copolymer. Thus, the molecular weight (*M*_n,NMR_) can be calculated to be 4731 g/mol by the equation *M*_n,NMR_ = (104 + 309) × (*I*_6.50–7.75_/10) + 271 = (104 + 309) × [(216.0/2)/10] + 271, which was close to the value obtained by GPC (*M*_n,GPC_ = 4600 g/mol). In ^13^C NMR of the copolymer (Figure 6a), signals at 173.2 ppm represented the C=O groups in maleimide units. Figure 3 showed the MALDI-TOF mass spectrum of polymer ([St]_0_/[MSM]_0_/[CPDN]_0_/[AIBN]_0_ = 200/200/2/1, precipitated in methanol, *M*_n_ = 4600 g/mol, *Ð* = 1.47). There were two kinds of peak serials with repeat units of 104.3 Da and 153.9 Da, which could be ascribed to the MW of St and MSM respectively. The determined value of 1082.4 Da was matched the theoretical value of 1083.4 Da, which was corresponded to the polymer structure containing 4 St units and 3 MSM units after the elimination of CPDN fragment at the chain end of copolymer during MALDI-TOF mass testing. Loss of the selenium-containing fragment was attributed to a weak selenium-carbon bond cleavage and removal from the copolymer during MALDI-TOF mass testing [31]. In addition, the copolymer was analyzed by FT-IR spectrum (Figure S7) which showed characteristic adsorption peaks according to carbon-oxygen double bond at 1690 cm^−1^. The UV-vis spectrum of copolymer ([St]_0_/[MSM]_0_/[CPDN]_0_/[AIBN]_0_ = 200/200/2/1, precipitated in methanol, *M*_n_ = 4600 g/mol, *Ð* = 1.47) before oxidation showed weak adsorption peak at around 310 nm, which can be attributed the adsorption of selenide moiety (Figure S8a). Such an adsorption peak disappeared in the UV-vis spectrum of copolymer after the oxidation (*M*_n_ = 2300 g/mol, *Ð* = 1.43) (Figure S8b) verified the elimination of selenium phenyl groups. Therefore, the above data confirmed that the selenium-containing monomer MSM was successfully copolymerized with St and got a controlled copolymer.

### 3.2. Oxidization of Copolymer

**Figure 4:**
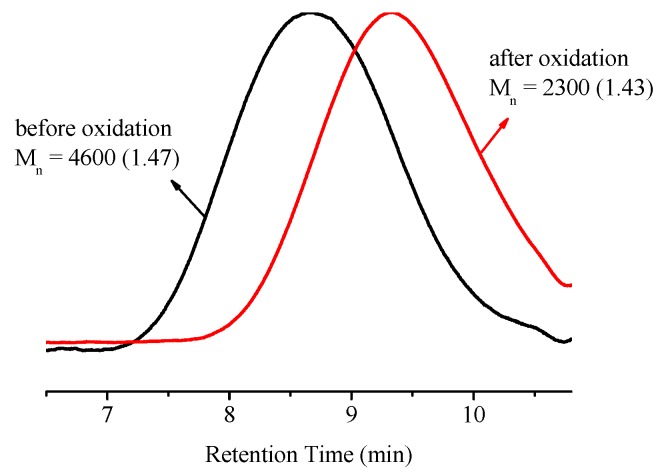
Gel permeation chromatograph (GPC) traces before and after oxidation using 30% H_2_O_2_.

**Figure 5:**
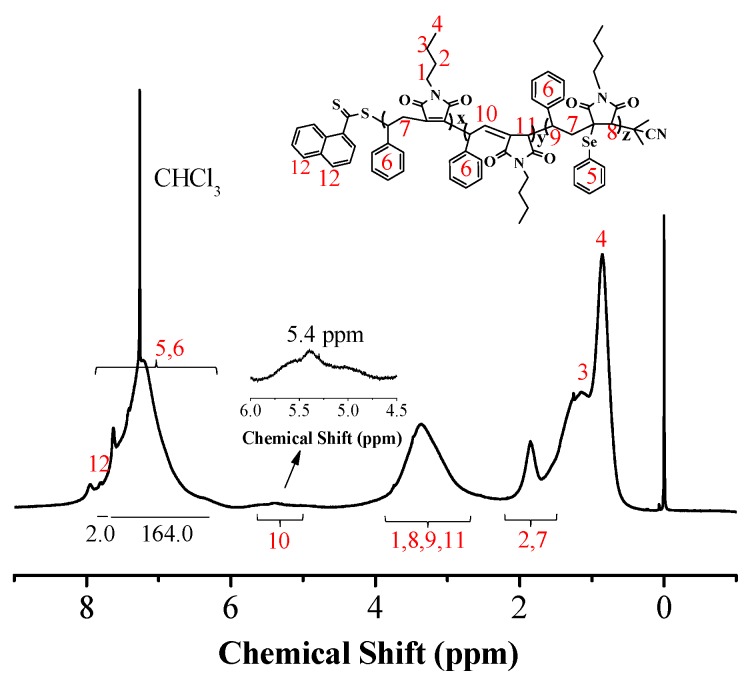
^1^H NMR spectrum of oxidized copolymer in CDCl_3_.

**Figure 6:**
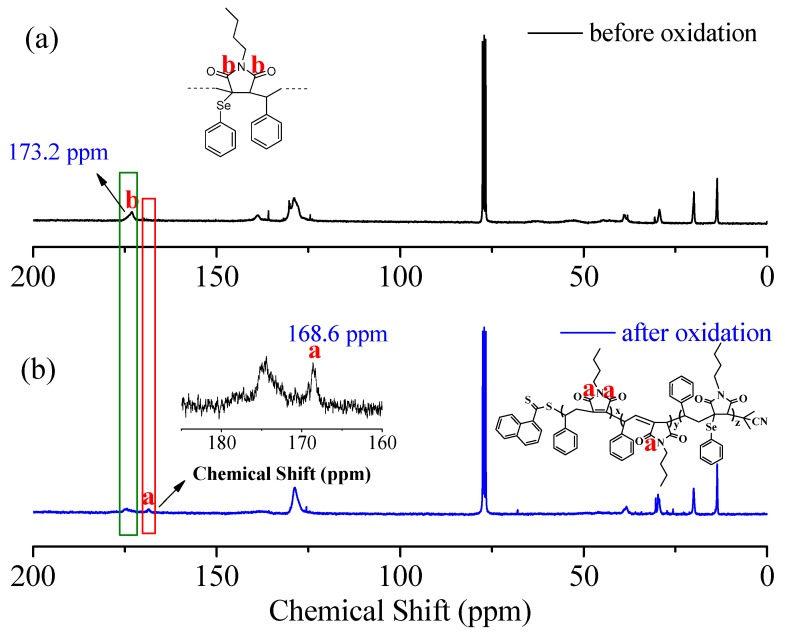
^13^C NMR spectrum of the oxidized copolymer.

The oxidative elimination of selenide moieties is one of the well-known reactions for selenium-containing compounds [32,33]. Herein, the obtained copolymer ([St]_0_/[MSM]_0_/[CPDN]_0_/[AIBN]_0_ = 200/200/2/1, precipitated in methanol, *M*_n_ = 4600 g/mol, *Ð* = 1.47) was treated with hydrogen peroxide (H_2_O_2_, 30%). The possible reaction was showed as Scheme 1. The structure of copolymer before and after the oxidization was characterized by GPC (Figure 4). It showed that the molecular weight of copolymer was decreased from 4600 to 2300 g/mol after oxidization. Such a molecular weight decrease of the copolymer could be ascribed to the cleavage of the weak selenium-carbon bond. The structure of the oxidized copolymer was investigated by ^1^H NMR spectrum as showed in Figure 5. In Figure 5, the oxidized copolymer showed the signals at about 4.80–5.80 ppm, which were ascribed to the protons from C=C double bonds. The signals at about 7.77–7.85 ppm (2H, integration = *I*_7.77–7.85_ = 2.0), which were ascribed to the protons at the chain end derived from CPDN, the signals at about 6.20–7.75 ppm (integration = *I*_6.20–7.75_ = 164.0) were ascribed to the protons of benzyl from repeat unites. Thus, combined with the ^1^H NMR of copolymer before oxidization, the oxidization yield can be calculated to be 48% by the equation oxidization yield = [(216.0/2 − 164.0/2))/5]/[(216.0/2)/10]. Such structure evolution also can be illustrated from the ^13^C NMR in Figure 6b. The signals for the C=O groups in the maleimide repeat unit shifted from 173.2 to 168.6 ppm due to the formation of electron rich carbon-carbon double bond adjacent to C=O after oxidation. However, the reaction efficiency of such a reaction in the current case was lower than those found in the literature [33,34]. However, the current results clearly demonstrated that the phenylselenide substitution could be partial oxidative cleavage from maleimide by 30% H_2_O_2_, which resulted in the introducing of carbon-carbon double bonds in the backbone of copolymer.

### 3.3. Thermal Properties of Selenium-Containing Copolymer and Oxidized Copolymer

**Figure 7:**
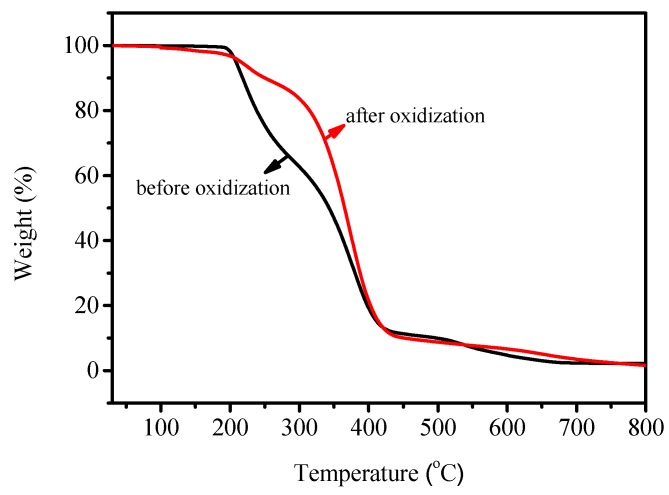
Thermogravimetric analysis (TGA) spectra of copolymers before and after oxidization.

**Figure 8:**
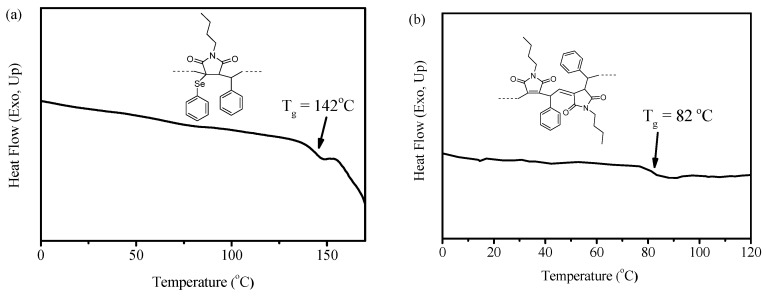
Differential scanning calorimetry (DSC) spectra of copolymers: (**a**) before oxidization (**b**) after oxidization.

To determine the decomposition temperature of the copolymer before and after oxidization, TGA was tested under N_2_ atmosphere. The results were summarized in Figure 7. The degradation temperature of copolymer before oxidization ([St]_0_/[MSM]_0_/[CPDN]_0_/[AIBN]_0_ = 200/200/2/1, precipitated in methanol, *M*_n_ = 4600 g/mol, *Ð* = 1.47) was found to be 208 °C, causing the elimination of phenyl-selenide groups. For oxidized copolymer, the first degradation step was found at around 130 °C, and its maximum weight loss occurred at 215 °C with a 5% total weight loss which could be assigned to the elimination of phenyl-selenide groups. This was expected because the copolymer without oxidization possesses thermal stability higher than its oxidized copolymer which has been reported to initiate even at room temperature and increase rapidly with temperature [35]. We employed DSC under N_2_ to examine the glass transition temperature (*T_g_*) of copolymer before and after the oxidation. The results were showed in Figure 8. It indicated that the *T_g_* of oxidized copolymer (82 °C) was lower than that of copolymer before oxidization (142 °C), which is presumably due to the introduction of a flexible double bond in the main chain after elimination of selenium phenyl groups.

### 3.4. Photoluminescence Investigation

**Figure 9:**
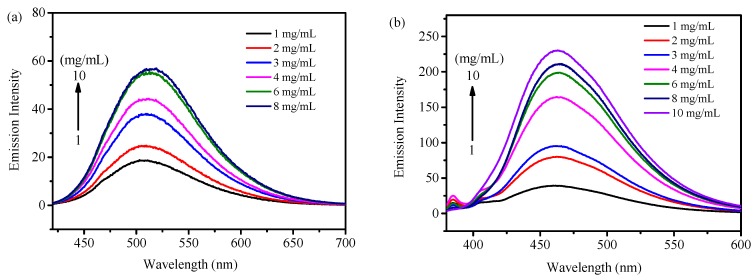
Photoluminescence (PL) spectra in THF at different concentrations (**a**) copolymer before oxidization, excited at 410 nm; (**b**) oxidized copolymer, excited at 370 nm.

**Figure 10:**
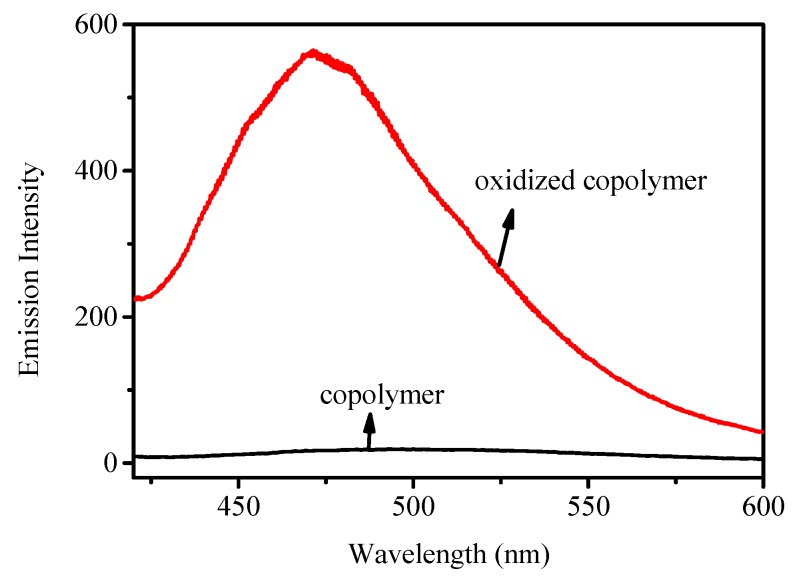
PL spectra of copolymer and oxidized copolymer at solid state, excited at 370 nm.

It was demonstrated in the literature that maleimide with difference substitution showed interesting photoluminescence (PL) behavior. Thus, the photoluminescence behavior of copolymer ([St]_0_/[MSM]_0_/[CPDN]_0_/[AIBN]_0_ = 200/200/2/1, precipitated in methanol, *M*_n_ = 4600 g/mol, *Ð* = 1.47) and its oxidized product (*M*_n_ = 2300 g/mol, *Ð* = 1.43) was investigated in solution and solid state. In THF, the copolymers before and after oxidization had excitation maxima at 410 nm and 370 nm respectively, see Figure S9. Figure 9 showed PL spectra of copolymer before and after oxidation in THF with different concentration. It showed that the PL behavior of copolymer in solution could be changed before and after the oxidation. The polymer before oxidation showed PL emission maximum at 510 nm with exciting wavelength of 410 nm. The PL emission maximum shifted to 460 nm after oxidation with exciting wavelength of 370 nm due to the elimination of phenyl selenide. Furthermore, the PL emission enhancement with concentration was found in both of copolymers before and after the oxidation, which may be due to the aggregation of carbonyl groups in maleimide repeat units [36]. It was clear that such enhancement with concentration was more sensitive in case of copolymer after oxidation, both with or without the internal heavy atom effects of selenium atoms [37,38]. Such an effect was more noticeable at solid state. In Figure 10, with excitation at 370 nm, the spectrum of oxidized copolymer at solid state featured strong emission peaks at 460 nm. In contrast, the spectrum of copolymer without oxidization at solid state featured hardly any signal because of the impact from selenium.

## 4. Conclusions

The novel selenium-containing maleimide monomer MSM was successfully copolymerized with St through RAFT copolymerization at 70 °C using CPDN as the chain transfer agent and AIBN as the initiator. The feature of the controlled copolymerization was proven by the linear kinetic plots, controlled molecular weight and narrow molecular weight distribution of the copolymer. The structure of the obtained copolymer could be modified by eliminating phenylselenide under oxidative condition. This strategy provides a new method to prepare copolymers with selenide functional groups available for carbon-carbon double bonds formation in the backbone of a copolymer. By such structural modification, the thermal stability and PL emission property could be adjusted, e.g., improved thermal stability and enhanced PL emission intensity with concentration.

## Figures and Tables

**Table 1:** Reversible addition-fragmentation chain transfer (RAFT) polymerization of St and monoselenomaleimide (MSM) under different molar ratios ^a^.

Entry	[St]_0_/[MSM]_0_/[CPDN]_0_/[AIBN]_0_	Time (h)	Conv._St_^b^ (%)	Conv._MSM_^b^ (%)	*M*_n_^c^ (g/mol)	*Đ* ^c^
1	200/6/2/1	2.0	20	100	2000	1.28
2	200/20/2/1	2.5	51	100	2600	1.35
3	200/60/2/1	10	27	25	2500	1.43
4	200/200/2/1	9.0	34	27	3100	1.62
5	0/200/2/1	24.0	N.A.	-	-	-

Polymerization conditions: ^a^ *V*_St_ = 1.0 mL, *V*_toluene_ = 50 µL, under argon protection, 70 °C; ^b^ Calculated from ^1^H NMR of the raw experimental samples by comparing the integration of one vinyl protons of remaining monomer to the integration of initial monomer with toluene as internal standard; ^c^ Determined by GPC with polystyrene as standards.

## Supplementary Materials

The following are available online at http://www.mdpi.com/2073-4360/10/3/321/s1, NMR spectra, GPC curves, FT-IR spectrum, and UV-vis spectra of polymers.
