# Supplementary material for: Copolymerization of Phenylselenide-Substituted Maleimide with Styrene and Its Oxidative Elimination Behavior
Source: Polymers (Basel). 2018 Mar 15;10(3):321. doi: 10.3390/polym10030321 (PMC6415178; doi:10.3390/polym10030321)
Supplement: Supplementary file 1 [file polymers-10-00321-s001.pdf]

Supporting Information for

Copolymerization of Phenylselenide Substituted Maleimide with Styrene and Its Oxidative Elimination Behavior

Qian Liu, Xinghua Lv, Na Li, Xiangqiang Pan*, Jian Zhu*, and Xiulin Zhu

State and Local Joint Engineering Laboratory for Novel Functional Polymeric Materials, Department of Polymer Science and Engineering, College of Chemistry, Chemical Engineering and Materials Science, Soochow University, Suzhou, 215123, China

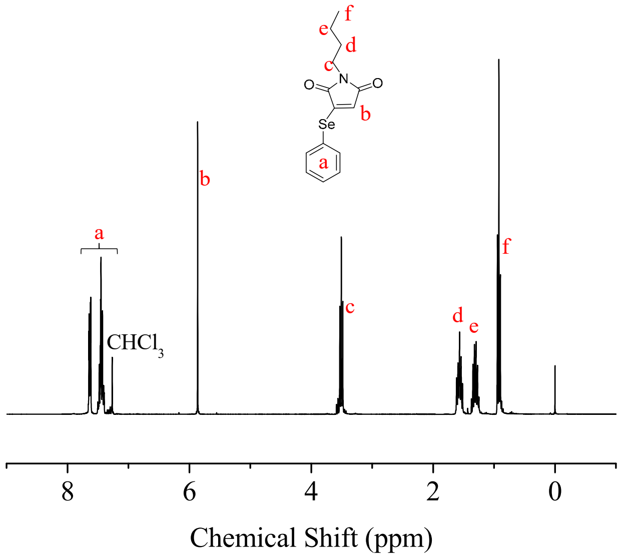

Figure S1. ^{1}H NMR spectrum of MSM in $CDCl_3$

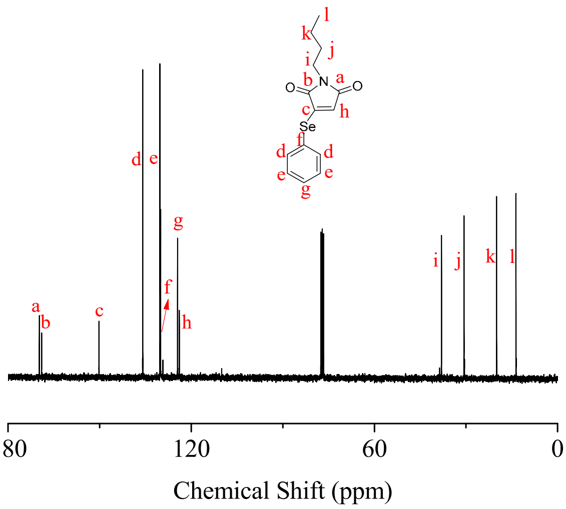

Figure S2. ^{13}C NMR spectrum of MSM in $CDCl_3$

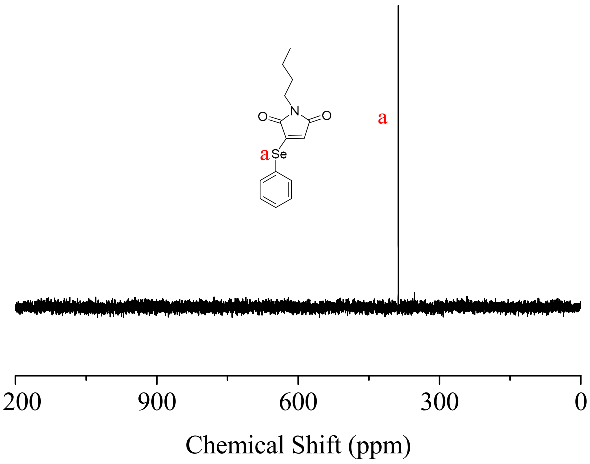

Figure S3. ^{77}Se NMR spectrum of MSM in $CDCl_3$

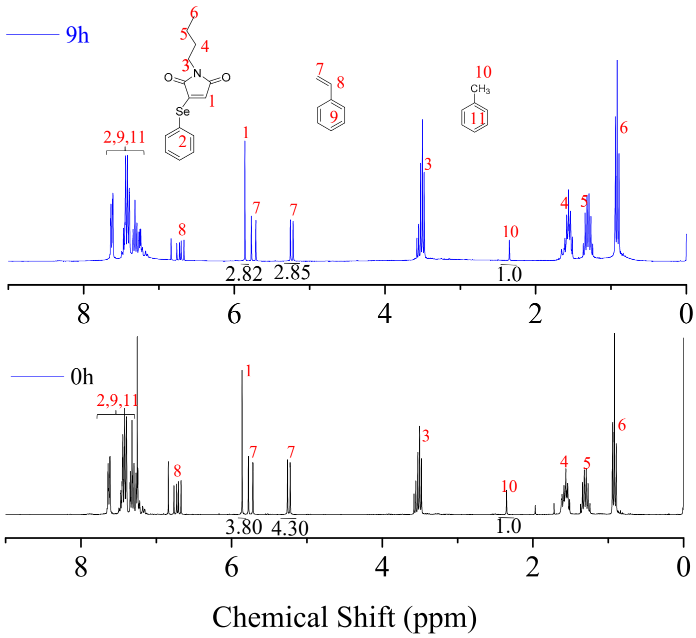

Figure S4. Direct ^{1}H NMR analysis spectra of monomer conversion in $CDCl_3$. The integral of the signal of proton belong to toluene in the ^{1}H NMR was set to be 1.0, served as internal standard for the decrease of the integral of double bonds belong to each monomer. 0 h and 9 h was toward initial time and the ninth hour of the whole copolymerization. $Conv._{St} = (I_{5.26,0h} - I_{5.26,9h}) / I_{5.26,0h} \times 100\%$; $Conv._{MSM} = (I_{5.86,0h} - I_{5.86,9h}) / I_{5.86,0h} \times 100\%$. The conversion of monomer at other times is similar to the calculation illustrated above.

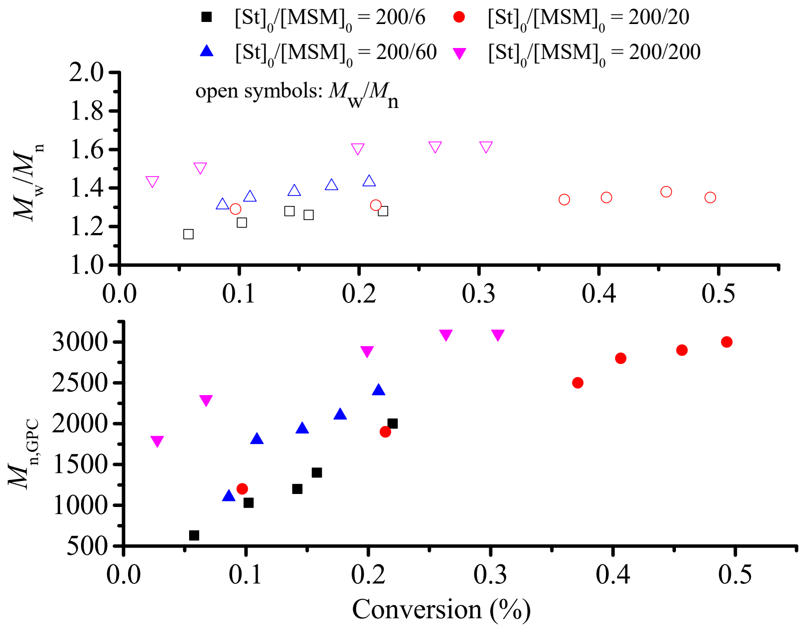

Figure S5. Relationships of M_n and molecular weight distribution (*Đ*) with conversion of reversible addition-fragmentation chain transfer (RAFT) copolymerization of St and MSM with different molar ratios

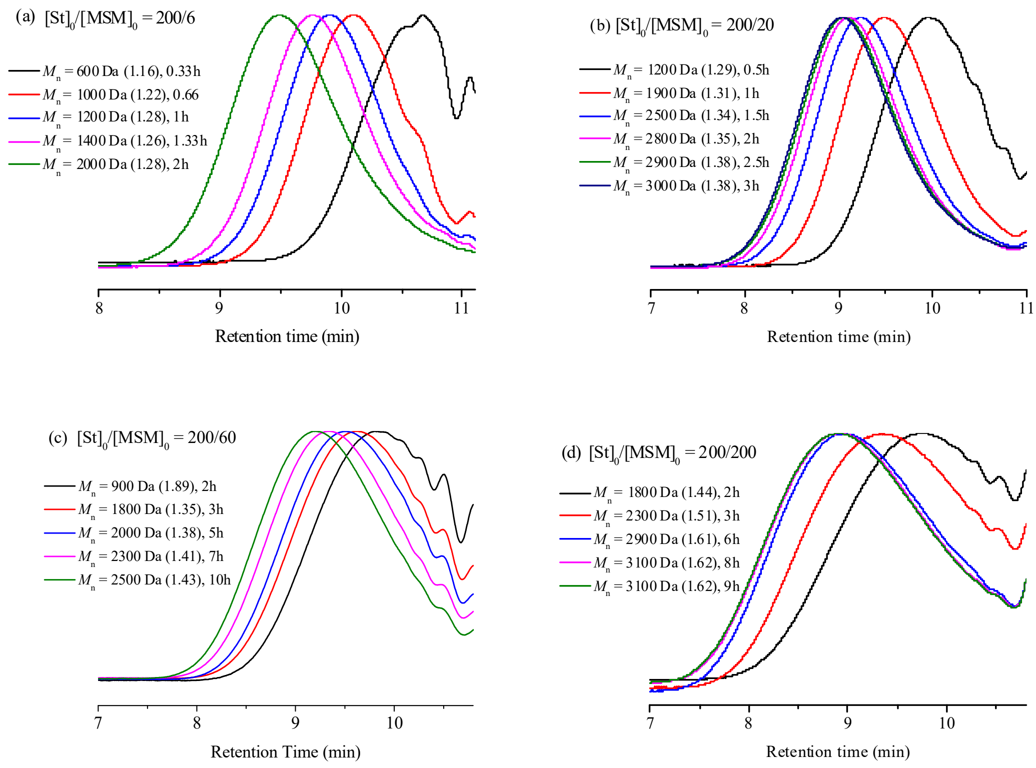

Figure S6. GPC curves of reversible addition-fragmentation chain transfer (RAFT) copolymerization of St and MSM with different molar ratios, (a) $[St]_0/[MSM]_0$ = 200/6; (b) $[St]_0/[MSM]_0$ = 200/20; (c) $[St]_0/[MSM]_0$ = 200/60; (d) $[St]_0/[MSM]_0$ = 200/200, V_{St} = 1.0 mL, $V_{toluene}$ = 50 µL, temperature 70 °C.

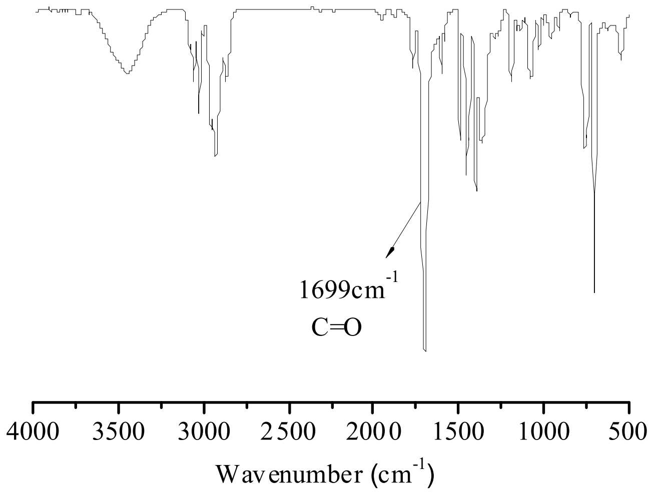

Figure S7. FT-IR spectrum of the RAFT copolymer

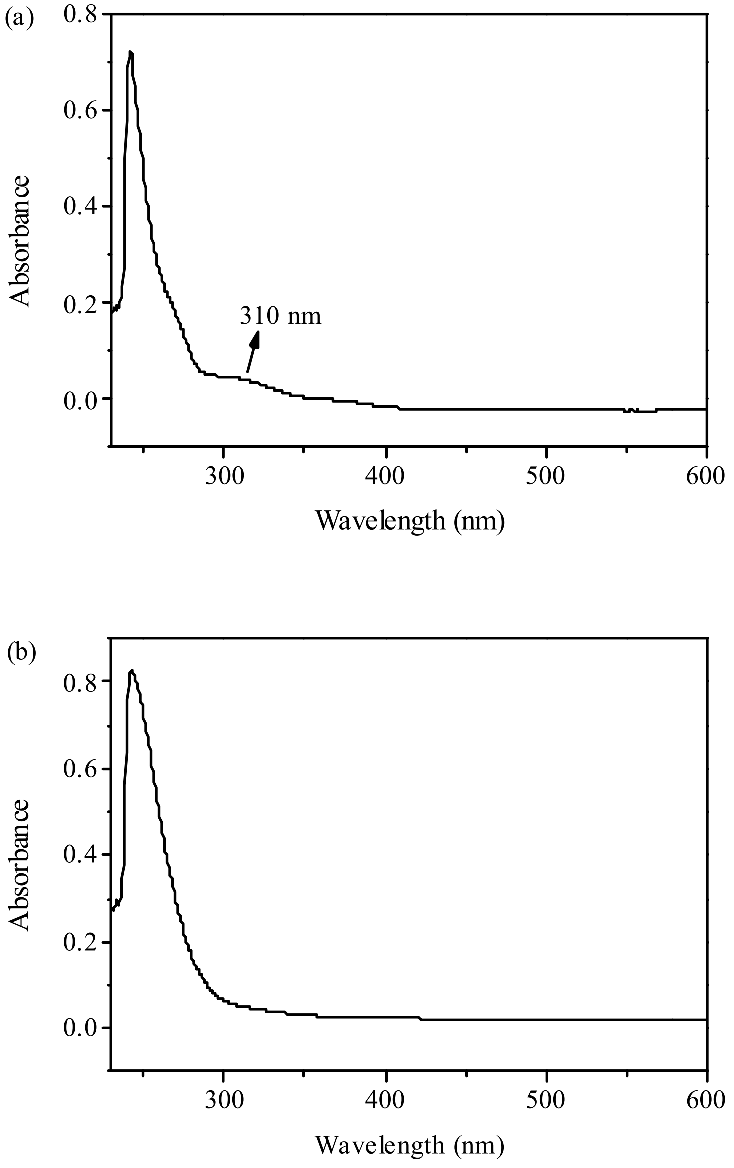

Figure S8. UV-*vis* spectra of copolymers in THF: (a) before oxidization (b) after oxidization

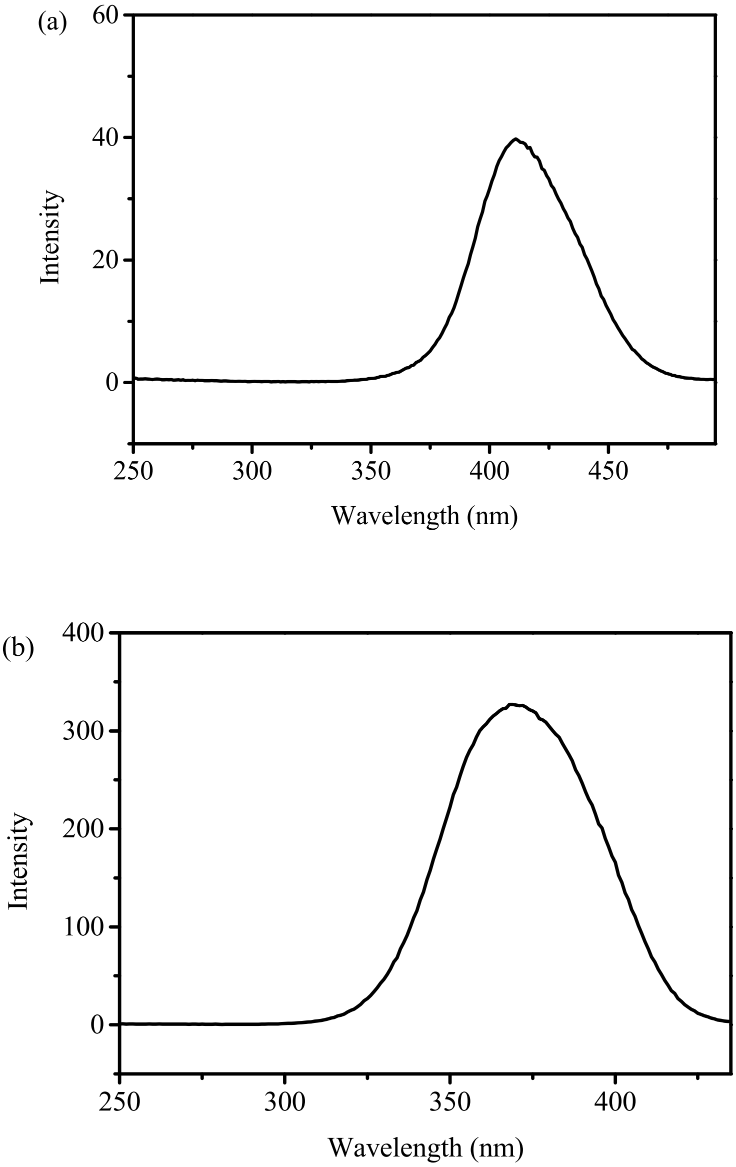

Figure S9. Excitation spectra of copolymers in THF at 10 mg/mL: (a) before oxidization (b) after oxidization
